# 4,4′-(1,2,4,5-Tetra­zine-3,6-di­yl)dibenzo­nitrile

**DOI:** 10.1107/S1600536809008599

**Published:** 2009-03-25

**Authors:** Grzegorz Dutkiewicz, Teresa Borowiak, Jarosław Spychała

**Affiliations:** a Department of Chemistry, Adam Mickiewicz University, Grunwaldzka 6, 60-780 Poznań, Poland

## Abstract

Mol­ecules of the title compound, C_16_H_8_N_6_, lie on crystallographic inversion centres. A dihedral angle of 16.1 (1)° is formed between the central tetra­zine ring and the plane of each cyano­phenyl group. The mol­ecules form stacks along [100] with a perpendicular inter­planar separation of 3.25 (1) Å. C—H⋯N inter­actions are formed between mol­ecules in neighbouring stacks.

## Related literature

For synthesis details, see: Spychała *et al.* (1994 ▶, 2000 ▶). For related structures and discussion, see: Higashi & Osaki (1981 ▶); Infantes *et al.* (2003 ▶).
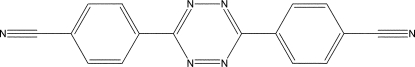


## Experimental

### 

#### Crystal data

C_16_H_8_N_6_
*M* *_r_* = 284.28
Monoclinic, 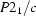

*a* = 4.8447 (5) Å
*b* = 12.1054 (12) Å
*c* = 11.6927 (11) Å
β = 94.363 (8)°
*V* = 683.75 (12) Å^3^
*Z* = 2
Mo *K*α radiation
μ = 0.09 mm^−1^
*T* = 291 K
0.45 × 0.2 × 0.1 mm

#### Data collection

Kuma KM-4-CCD diffractometer
Absorption correction: multi-scan (*CrysAlis RED*; Oxford Diffraction, 2007 ▶) *T*_min_ = 0.925, *T*_max_ = 0.991
5912 measured reflections
1768 independent reflections
1094 reflections with *I* > 2σ(*I*)
*R*_int_ = 0.017

#### Refinement

*R*[*F* ^2^ > 2σ(*F* ^2^)] = 0.044
*wR*(*F* ^2^) = 0.135
*S* = 1.06
1768 reflections
116 parameters
All H-atom parameters refined
Δρ_max_ = 0.16 e Å^−3^
Δρ_min_ = −0.13 e Å^−3^

### 

Data collection: *CrysAlis CCD* (Oxford Diffraction, 2007 ▶); cell refinement: *CrysAlis RED* (Oxford Diffraction, 2007 ▶); data reduction: *CrysAlis RED*; program(s) used to solve structure: *SHELXS97* (Sheldrick, 2008 ▶); program(s) used to refine structure: *SHELXL97* (Sheldrick, 2008 ▶); molecular graphics: *Stereochemical Workstation Operation Manual* (Siemens, 1989 ▶) and *Mercury* (Macrae *et al.*, 2006 ▶); software used to prepare material for publication: *SHELXL97*.

## Supplementary Material

Crystal structure: contains datablocks I, global. DOI: 10.1107/S1600536809008599/bi2354sup1.cif

Structure factors: contains datablocks I. DOI: 10.1107/S1600536809008599/bi2354Isup2.hkl

Additional supplementary materials:  crystallographic information; 3D view; checkCIF report

## Figures and Tables

**Table 1:** Hydrogen-bond geometry (Å, °)

*D*—H⋯*A*	*D*—H	H⋯*A*	*D*⋯*A*	*D*—H⋯*A*
C6—H6⋯N10^i^	0.989 (17)	2.539 (17)	3.370 (2)	141.6 (13)
C8—H8⋯N2^ii^	0.956 (17)	2.850 (17)	3.6106 (19)	137.2 (12)
C9—H9⋯N10^iii^	0.993 (17)	2.754 (17)	3.431 (2)	125.8 (12)

Symmetry codes: (i) 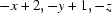; (ii) 
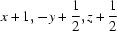
; (iii) 
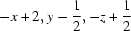
.

**Fig. 1.**
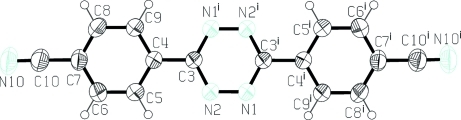
Molecular structure showing displacement ellipsoids at the 50% probability level for non-H atoms. Symmetry code: (i) -x, -y, -z.

**Fig. 2.**
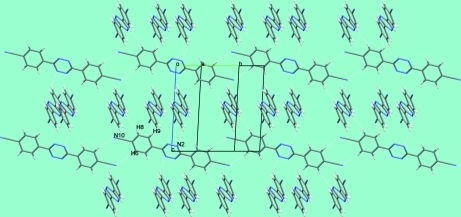
Chains of molecules (horizontal) linked by centrosymmetric pairs of C—H···N(cyano) interactions.

**Fig. 3.**
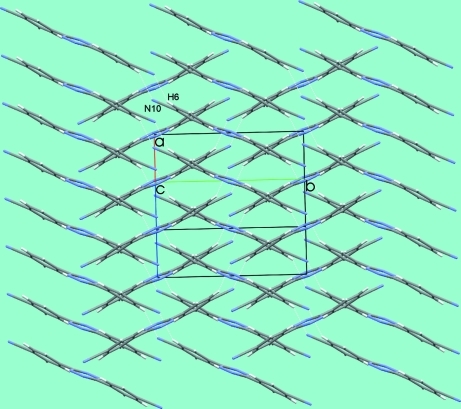
Stacks of molecules (vertical) showing the "stepped" arrangement within the 1-D chains.

## supplementary crystallographic information

### Comment

Infantes *et al.* (2003) have found that the supramolecular structures of some substituted phenyl derivatives of 1,2,4,5-tetrazine are comparable to those of their carboxylic acid analogues. Being inspired by that, we have compared the supramolecular structures of the title compound 3,6-bis(4-cyanophenyl)-1,2,4,5-tetrazine (hereafter I) and *p*-cyanobenzoic acid (Higashi & Osaki, 1981) (hereafter II).

In (I), the tetrazine molecule is located on a crystallographic inversion centre (Fig. 1). The phenyl rings are twisted with respect to the tetrazine ring by 16.1 (1)° in opposite directions. The cyano-groups are coplanar with their phenyl rings. Two C6—H6···N10(cyano) interactions related by a centre of inversion can be considered to link the molecules into 1-D chains (Fig. 2). The chains are "stepped" rather than flat (Fig. 3). Each molecule interacts with the neighbouring chain through C8—H8···N2(tetrazine) and C9—H9···N10(cyano) interactions (Fig. 2), and the molecules are stacked along [100] with a perpendicular interplanar spacing of 3.25 (1) Å. This structure contrasts with the layered structures of other phenyl-derivatives of 1,2,4,5 tetrazines described in the paper by Infantes *et al.* (2003).

In the crystal structure of (II), similar 1-D chains are formed through the well-known centrosymmetric carboxylic acid dimer on one side of the molecule and centrosymmetric C—H···N(cyano) interactions on the other side of the molecule. The latter interactions are closely comparable to those in (I), except that the chains in (II) lie in approximately flat layers parallel to the (201) planes. The distinction between the two structures arises because of differences between the lateral C—H···O interactions between chains in (II) and the C—H···N(tetrazine) and C—H···N(cyano) interactions in (I).

### Experimental

The title compound was obtained from a multi-step procedure starting from 4-amidinobenzamide hydrochloride and anhydrous hydrazine. Dehydration of the biscarbamoyl intermediate compound to the appropriate biscyano red product was effected by means of phosphorus oxychloride in the same way as described for 2,4-bis(4- carbamoylphenyl)-1,3,5-triazine (Spychała *et al.*, 1994; Spychała (2000). M.p. 568–570 K (acetone); δ_H_ (CDCl_3_, TMS) 7.94 (d, 4H, *J* = 8.8 Hz, CH), 8.82 (d, 4H, *J* = 8.8 Hz, CH); δ_C_ (DMSO-d_6_) 114.7, 117.8, 128.2, 133.0, 135.6, 162.4; MS (EI) 284 (*M*^+^, C_16_H_8_N_6_; 13), 128 (100), 102 (9), 101 (33), 100 (7), 77 (9), 76 (12), 75 (16), 74 (4), 64 (11).

Single crystals were grown from hot acetone by slow cooling.

### Refinement

All H atoms were found from difference Fourier maps and refined freely with isotropic displacement parameters.

### Crystal data

**Table:**

C_16_H_8_N_6_	*F*(000) = 292
*M**_r_* = 284.28	*D*_x_ = 1.381 Mg m^−^^3^
Monoclinic, *P*2_1_/*c*	Mo *K*α radiation, λ = 0.71073 Å
Hall symbol: -P 2ybc	Cell parameters from 2059 reflections
*a* = 4.8447 (5) Å	θ = 2.4–29.6°
*b* = 12.1054 (12) Å	µ = 0.09 mm^−^^1^
*c* = 11.6927 (11) Å	*T* = 291 K
β = 94.363 (8)°	Block, orange
*V* = 683.75 (12) Å^3^	0.45 × 0.2 × 0.1 mm
*Z* = 2	

### Data collection

**Table:**

Kuma KM-4-CCD diffractometer	1768 independent reflections
Radiation source: fine-focus sealed tube	1094 reflections with *I* > 2σ(*I*)
graphite	*R*_int_ = 0.017
Detector resolution: 8.1929 pixels mm^-1^	θ_max_ = 29.7°, θ_min_ = 3.4°
ω scans	*h* = −6→6
Absorption correction: multi-scan (*CrysAlis RED*; Oxford Diffraction, 2007)	*k* = −16→15
*T*_min_ = 0.925, *T*_max_ = 0.991	*l* = −15→14
5912 measured reflections	

### Refinement

**Table:**

Refinement on *F*^2^	Primary atom site location: structure-invariant direct methods
Least-squares matrix: full	Secondary atom site location: difference Fourier map
*R*[*F*^2^ > 2σ(*F*^2^)] = 0.044	Hydrogen site location: difference Fourier map
*wR*(*F*^2^) = 0.135	All H-atom parameters refined
*S* = 1.06	*w* = 1/[σ^2^(*F*_o_^2^) + (0.0683*P*)^2^ + 0.0311*P*] where *P* = (*F*_o_^2^ + 2*F*_c_^2^)/3
1768 reflections	(Δ/σ)_max_ < 0.001
116 parameters	Δρ_max_ = 0.16 e Å^−^^3^
0 restraints	Δρ_min_ = −0.13 e Å^−^^3^

### Special details

**Table:**

Geometry. All e.s.d.'s (except the e.s.d. in the dihedral angle between two l.s. planes) are estimated using the full covariance matrix. The cell e.s.d.'s are taken into account individually in the estimation of e.s.d.'s in distances, angles and torsion angles; correlations between e.s.d.'s in cell parameters are only used when they are defined by crystal symmetry. An approximate (isotropic) treatment of cell e.s.d.'s is used for estimating e.s.d.'s involving l.s. planes.
Refinement. Refinement of *F*^2^ against ALL reflections. The weighted *R*-factor *wR* and goodness of fit *S* are based on *F*^2^, conventional *R*-factors *R* are based on *F*, with *F* set to zero for negative *F*^2^. The threshold expression of *F*^2^ > σ(*F*^2^) is used only for calculating *R*-factors(gt) *etc*. and is not relevant to the choice of reflections for refinement. *R*-factors based on *F*^2^ are statistically about twice as large as those based on *F*, and *R*- factors based on ALL data will be even larger.

### Fractional atomic coordinates and isotropic or equivalent isotropic displacement parameters (Å^2^)

**Table:**

	*x*	*y*	*z*	*U*_iso_*/*U*_eq_	
N1	−0.2003 (2)	0.01444 (9)	−0.08623 (10)	0.0532 (4)	
N2	−0.0256 (2)	0.09630 (9)	−0.05942 (10)	0.0528 (4)	
C3	0.1693 (2)	0.07922 (10)	0.02639 (10)	0.0418 (3)	
C4	0.3614 (2)	0.17078 (10)	0.05676 (11)	0.0434 (3)	
C5	0.3849 (3)	0.25828 (12)	−0.01845 (13)	0.0546 (4)	
C6	0.5664 (3)	0.34327 (13)	0.00878 (14)	0.0596 (4)	
C7	0.7235 (3)	0.34205 (12)	0.11308 (13)	0.0542 (4)	
C8	0.7010 (3)	0.25569 (14)	0.18883 (14)	0.0626 (5)	
C9	0.5218 (3)	0.16937 (13)	0.16039 (13)	0.0564 (4)	
C10	0.9104 (3)	0.43269 (15)	0.14189 (14)	0.0682 (5)	
N10	1.0547 (4)	0.50498 (14)	0.16340 (14)	0.0986 (6)	
H6	0.588 (3)	0.4047 (13)	−0.0455 (16)	0.080 (5)*	
H5	0.271 (3)	0.2606 (13)	−0.0895 (14)	0.065 (4)*	
H8	0.811 (3)	0.2535 (13)	0.2601 (15)	0.078 (5)*	
H9	0.509 (3)	0.1054 (14)	0.2128 (14)	0.076 (5)*	

### Atomic displacement parameters (Å^2^)

**Table:**

	*U*^11^	*U*^22^	*U*^33^	*U*^12^	*U*^13^	*U*^23^
N1	0.0548 (7)	0.0474 (7)	0.0549 (7)	−0.0081 (5)	−0.0131 (5)	0.0051 (5)
N2	0.0546 (7)	0.0469 (7)	0.0542 (7)	−0.0070 (5)	−0.0128 (5)	0.0056 (5)
C3	0.0429 (7)	0.0433 (7)	0.0388 (7)	−0.0019 (6)	0.0003 (5)	−0.0008 (5)
C4	0.0423 (7)	0.0441 (7)	0.0433 (7)	−0.0026 (6)	−0.0005 (5)	−0.0012 (6)
C5	0.0578 (8)	0.0545 (9)	0.0496 (8)	−0.0100 (7)	−0.0075 (6)	0.0061 (7)
C6	0.0670 (10)	0.0537 (9)	0.0572 (9)	−0.0152 (8)	−0.0005 (7)	0.0056 (7)
C7	0.0534 (8)	0.0538 (9)	0.0553 (9)	−0.0146 (7)	0.0033 (6)	−0.0068 (7)
C8	0.0640 (9)	0.0719 (10)	0.0494 (8)	−0.0190 (8)	−0.0114 (7)	0.0007 (8)
C9	0.0620 (9)	0.0566 (9)	0.0487 (8)	−0.0149 (7)	−0.0086 (7)	0.0060 (7)
C10	0.0749 (10)	0.0741 (11)	0.0554 (9)	−0.0259 (9)	0.0034 (8)	−0.0063 (8)
N10	0.1182 (13)	0.1029 (13)	0.0742 (11)	−0.0672 (11)	0.0037 (9)	−0.0105 (9)

### Geometric parameters (Å, °)

**Table:**

N1—N2	1.3254 (14)	C6—C7	1.388 (2)
N1—C3^i^	1.3347 (17)	C6—H6	0.989 (17)
N2—C3	1.3403 (17)	C7—C8	1.380 (2)
C3—N1^i^	1.3347 (17)	C7—C10	1.446 (2)
C3—C4	1.4735 (17)	C8—C9	1.383 (2)
C4—C5	1.3869 (19)	C8—H8	0.955 (17)
C4—C9	1.3888 (18)	C9—H9	0.993 (17)
C5—C6	1.375 (2)	C10—N10	1.1360 (18)
C5—H5	0.962 (16)		
			
N2—N1—C3^i^	117.81 (11)	C5—C6—H6	120.8 (10)
N1—N2—C3	117.55 (11)	C7—C6—H6	119.6 (10)
N1^i^—C3—N2	124.64 (11)	C8—C7—C6	120.49 (13)
N1^i^—C3—C4	117.94 (11)	C8—C7—C10	120.27 (13)
N2—C3—C4	117.42 (11)	C6—C7—C10	119.25 (14)
C5—C4—C9	119.68 (12)	C7—C8—C9	119.80 (14)
C5—C4—C3	120.17 (12)	C7—C8—H8	121.0 (10)
C9—C4—C3	120.15 (12)	C9—C8—H8	119.1 (10)
C6—C5—C4	120.43 (13)	C8—C9—C4	119.99 (14)
C6—C5—H5	119.6 (9)	C8—C9—H9	120.7 (10)
C4—C5—H5	120.0 (9)	C4—C9—H9	119.4 (10)
C5—C6—C7	119.60 (14)	N10—C10—C7	178.9 (2)
			
C3^i^—N1—N2—C3	−0.3 (2)	C4—C5—C6—C7	−1.0 (2)
N1—N2—C3—N1^i^	0.3 (2)	C5—C6—C7—C8	0.7 (2)
N1—N2—C3—C4	−179.39 (11)	C5—C6—C7—C10	−178.94 (15)
N1^i^—C3—C4—C5	164.01 (13)	C6—C7—C8—C9	0.4 (3)
N2—C3—C4—C5	−16.25 (19)	C10—C7—C8—C9	−179.95 (15)
N1^i^—C3—C4—C9	−15.40 (19)	C7—C8—C9—C4	−1.2 (3)
N2—C3—C4—C9	164.34 (13)	C5—C4—C9—C8	0.9 (2)
C9—C4—C5—C6	0.2 (2)	C3—C4—C9—C8	−179.71 (14)
C3—C4—C5—C6	−179.18 (13)		

Symmetry codes: (i) −*x*, −*y*, −*z*.

### Hydrogen-bond geometry (Å, °)

**Table:**

*D*—H···*A*	*D*—H	H···*A*	*D*···*A*	*D*—H···*A*
C6—H6···N10^ii^	0.989 (17)	2.539 (17)	3.370 (2)	141.6 (13)
C8—H8···N2^iii^	0.956 (17)	2.850 (17)	3.6106 (19)	137.2 (12)
C9—H9···N10^iv^	0.993 (17)	2.754 (17)	3.431 (2)	125.8 (12)

Symmetry codes: (ii) −*x*+2, −*y*+1, −*z*; (iii) *x*+1, −*y*+1/2, *z*+1/2; (iv) −*x*+2, *y*−1/2, −*z*+1/2.
